# Phenol Is the Initial Product Formed during Growth and Degradation of Bromobenzene by Tropical Marine Yeast, *Yarrowia lipolytica* NCIM 3589 via an Early Dehalogenation Step

**DOI:** 10.3389/fmicb.2017.01165

**Published:** 2017-06-23

**Authors:** Aakanksha A. Vatsal, Smita S. Zinjarde, Ameeta RaviKumar

**Affiliations:** Biochemistry Research Laboratory, Institute of Bioinformatics and Biotechnology, Savitribai Phule Pune UniversityPune, India

**Keywords:** bromobenzene, *Y. lipolytica*, degradation, dehalogenation, phenol, cell surface properties

## Abstract

Bromobenzene (BrB), a hydrophobic, recalcitrant organic compound, is listed by the environmental protection agencies as an environmental and marine pollutant having hepatotoxic, mutagenic, teratogenic, and carcinogenic effects. The tropical marine yeast *Yarrowia lipolytica* 3589 was seen to grow aerobically on BrB and displayed a maximum growth rate (μ_max_) of 0.04 h^-1^. Furthermore, we also observed an increase in cell size and sedimentation velocity for the cells grown on BrB as compared to the glucose grown cells. The cells attached to the hydrophobic bromobenzene droplets through its hydrophobic and acid–base interactions. The BrB (0.5%, 47.6 mM) was utilized by the cells with the release of a corresponding amount of bromide (12.87 mM) and yielded a cell mass of 1.86 g/L after showing 34% degradation in 96 h. Maximum dehalogenase activity of 16.16 U/mL was seen in the cell free supernatant after 24 h of growth. Identification of metabolites formed as a result of BrB degradation, namely, phenol, catechol, *cis, cis* muconic acid, and carbon dioxide were determined by LC–MS and GC–MS. The initial attack on bromobenzene by *Y. lipolytica* cells lead to the transient accumulation of phenol as an early intermediate which is being reported for the first time. Degradation of phenol led to catechol which was degraded by the ortho- cleavage pathway forming *cis, cis* muconic acid and then to Krebs cycle intermediates eventually leading to CO_2_ production. The study shows that dehalogenation via an extracellular dehalogenase occurs prior to ring cleavage with phenol as the preliminary degradative compound being produced. The yeast was also able to grow on the degradative products, i.e., phenol and catechol, to varying degrees which would be of potential relevance in the degradation and remediation of xenobiotic environmental bromoaromatic pollutants such as bromobenzene.

## Introduction

The widespread use of bromoaromatic compounds as flame retardants, pesticides, dyes or rubber additives and intermediates in the polymer industry (DePierre, 2003), has led to their accumulation in the environment. Their chemical inertness and hydrophobicity as well as their persistence and toxicity, has led to a severe concern about their environmental fate (Puzyn et al., 2011). Though bromoaromatics form a significant percentage of the halogenated compounds used currently, they have received far less attention as environmental pollutants as compared to their chlorinated analogs. A few reports exist on the anaerobic and aerobic bacterial degradation of bromoaromatics such as bromophenols, bromobenzoic acid (Commandeur and Parsons, 1990), polybromodiphenyl ethers (PBDPEs), tetrabromobisphenol A (TBBPA) and brominated flame retardants (BFRs) (Eljarrat et al., 2011) by *Azotobacter* sp. GP1, *Pseudomonas* sp., and *Rhodococcus* sp. However, there is little information on the fate and degradation of bromobenzene (United States Environmental Protection Agency [USEPA], 2009) by either bacterial and/or fungal systems although biodegradation of chloro- and fluorobenzenes has been documented previously (Allard and Nielson, 2003; Strunk and Engesser, 2013; Kiel and Engesser, 2015).

Bromobenzene (BrB) is a poorly soluble, hydrophobic organic compound listed as a priority environmental and marine pollutant and is a known hepatotoxic agent apart from having mutagenic, teratogenic, and carcinogenic effects (Darnerud, 2003; DePierre, 2003). It is used for synthesis in the production of phenyl magnesium bromide, additive in the motor oils and as a solvent in crystallization. The low bioavailability of BrB, due to its halogenated and hydrophobic state, has resulted in a decrease in its utilization and degradation by the microbes leading to its recalcitrance and bioaccumulation (United States Environmental Protection Agency [USEPA], 2009).

Microbial biodegradative routes of monochloro- and fluorobenzenes have been studied previously. Several microorganisms that can grow and utilize halobenzenes are able to metabolize these xenobiotic compounds to their corresponding halocatechols. Dehalogenation of the halobenzenes occurs after ring cleavage. Extensive studies on degradation of chlorobenzenes (Spain and Nishino, 1987; Spiess et al., 1995) and polychlorinated biphenyls (Macedo et al., 2007) by various bacterial strains have shown that they are degraded aerobically either by an early or late elimination of the chloride substituent involving dioxygenases and dehydrogenases. Early elimination, i.e., prior to aromatic ring cleavage leads to catechol formation which is degraded via the catechol pathway, whereas late elimination results in the chlorocatechol formation, which can be then further degraded by the modified ortho pathway, where the dehalogenation occurs during the metabolism of the ring-cleavage products (Reineke, 2001; Pieper et al., 2010). Yeast and fungi, generally degrade haloaromatic compounds co-metabolically via the ortho pathway to the corresponding halocatechol, which is then cleaved to the halogenated muconic acid. For example, *Penicillium frequentans* Bi 7/2 was shown to be capable of metabolizing various mono and dihalogenated phenols by oxidation to their corresponding halocatechols, while the basidiomycetous fungi use laccases, manganese, and lignin peroxidases to degrade haloaromatics (Hofrichter et al., 1994).

The marine ecosystem contains large amounts of bromoaromatic compounds which could be of natural or anthropogenic origin. As these compounds have been deposited into the oceans over a period in time, the marine microbes could have adapted to them and would be able to utilize these brominated xenobiotics. Such microbes are thus important in the bioremediation of polluted soil, groundwater, and wastewater. A majority of the microorganisms which degrade hydrophobic compounds do so by producing bio-active surfactants, which facilitate the uptake of these compounds increasing their bioavailability (Fickers et al., 2005). The past few years have seen the emergence of non-conventional yeasts such as *Yarrowia lipolytica*, which play an important role in the bioremediation of numerous industrial and environmental waste compounds (Bankar et al., 2009). A well-known property of *Y. lipolytica* is the ability to adhere and degrade hydrophobic substrates such as oils, *n*-alkanes and bromoalkanes (Fickers et al., 2005; Vatsal et al., 2011, 2015). We report here studies on *Y. lipolytica* NCIM 3589, which has been isolated from the marine water samples collected from areas near the offshore oil rig Mumbai High, contaminated with crude oil and industrial effluents. In fact, the Central Pollution Control Board of India [CPCB] (2008–2009,^2014–2015^) has also detected the presence of BrB in the industrial effluent belt along the western coast of India. As *Y. lipolytica* 3589 was isolated from an oil polluted zone and able to tolerate the harsh environmental conditions, it was anticipated to have the potential for utilizing brominated compounds for growth. In our earlier studies, we have reported the ability of this yeast to utilize and grow on brominated aliphatic compounds having different carbon chain lengths and different position and degree of the substituent bromide group (Vatsal et al., 2011, 2015). The growth kinetics would differ on the bromoaromatics present in the environment, depending on their bioavailability and the inherent capacity of the yeast to degrade them.

In this study, we report for the first time the growth on BrB and its degradation by the tropical marine yeast, *Y. lipolytica* NCIM 3589, under aerobic conditions. We also determine the degradative products formed wherein phenol is initially formed by a dehalogenation step, which is also being reported for the first time via a microbial degradation. Finally, the cell surface properties of *Y. lipolytica* 3589 were investigated in order to elucidate a probable mechanism by which it could be utilizing BrB.

## Materials and Methods

### Chemicals

The brominated aromatic compounds have been procured from Sisco Research Laboratories, India and Himedia Chemicals, India, while the solvents and media components were procured from Merck India, Ltd. All the chemicals were of AR grade, with an approximate 98% purity.

### Maintenance and Growth of Yeast Cells

Two strains of *Y. lipolytica*, NCIM 3589 and NCIM 3590 were used in this study. Stock cultures of the strains were maintained on MGYP plates (%, w/v) (0.3, malt extract; 1.0, glucose; 0.3, yeast extract; 0.5, peptone; 2.5, agar) and the yeast has been sub-cultured every month.

Flasks containing liquid minimal medium (MMY, %, pH 7.4) [0.54, Na_2_HPO_4_.12H_2_O; 0.14, KH_2_PO_4_; 0.05, (NH_4_)_2_SO_4_; 0.02, MgSO_4_. 7H_2_O; 0.001, yeast extract; 0.5% (v/v) salts solution and 0.5% (v/v) filter-sterilized vitamin solution] (Janssen et al., 1984; Vatsal et al., 2015) were used. For growth kinetics, *Y. lipolytica* NCIM 3589 cells (2 × 10^9^) were inoculated in 250 mL bottles containing MMY medium (50 mL) along with varying BrB concentrations (0.1–2.0%, v/v; 9.5–190 mM) and were sealed with Teflon-lined screw caps. These cells were allowed to grow aerobically at 30°C with a shaking speed of 150 rpm for a time period of 72 h. After growth, cells were centrifuged; pellet was washed with autoclaved distilled water, vortexed for separating the cells and absorbance measured at A_600_, wherein 1 OD was equivalent to 2 × 10^9^ cells/mL (Vatsal et al., 2011). The growth kinetic parameters were determined based on the relationship between the initial concentration of BrB and the specific growth rate and calculated by non-linear regression using Microcal Origin 6.0.

To investigate the effect of BrB concentration on growth of the cells, batch experiments were carried out. Initially, the yeast cells were grown on 1% MMYG [MMY containing 1% (w/v) of glucose] for a time period of 24 h to obtain biomass. The yeast cells were centrifuged at 4°C for 10 min at a speed of 7000 rpm. Then, the pellet was washed three times with sterile distilled water, vortexed, and absorbance measured at 600 nm. The cells were then collected and dried at 60°C to obtain a stable cell dry weight, which was estimated and a calibration curve for the optical density vs. dry weight plotted. 1 OD cells were inoculated with MMY medium (50 mL) in 250-mL Erlenmeyer flasks, containing BrB as the sole carbon source at a 0.5% v/v (47.6 mM) concentration till 96 h. Thereafter, the inoculated flasks were sealed for decreasing the abiotic loss of the volatile compounds. At a time interval of 24 h, one flask was collected and the cells were centrifuged at 7000 rpm at 4°C for 10 min. The cell free supernatant (CFS) obtained was used for estimating bromide ions and dehalogenase activity as described below. The residual concentration of BrB was determined by collecting the sample at the end of every time interval and the cell-free broth extracted as mentioned below. For determining the cell growth, the cells were vortexed for separating them from the hydrophobic droplets of BrB, then centrifuged and the pellet obtained was washed thrice with distilled water (sterile) and dried, and the dry weight was determined (Vatsal et al., 2011).

The cell growth on the metabolites of phenol and catechol (0.5, 1, 2.5, and 5 mM) in the MMY media was also determined after growing the yeast cells in aerobic conditions for 48 h and then dry weight determined.

### Light Microscopy Studies

For light microscopy studies, wet mounts of the washed yeast cells were prepared and carried out using a Zeiss microscope (Axioskope A1) with a camera and images were acquired, as described in Vatsal et al. (2011). The surface area was determined as *πd^2^* after assuming that the yeast cells were spherical. Though it is recognized that the individual yeast cells are not exactly spherical, the estimated surface area provides a relative index if there has been a change in the cell surface area after growing them on BrB.

The sedimentation velocity of the yeast cells was estimated using the Stokes law:

(1) $$Vs=\frac{D^{2}\times g\times \Delta \rho }{18\times \eta }$$

where, D is the cell diameter, g (gravity) is 9.807 m/s^2^, Δρ (cell density minus water density) is 100 kg/m^3^ and η (viscosity of water) is 9.04 × 10^-4^ kg/ms (Mortensen et al., 2005).

### Determination of the Cell Surface Properties

The yeast cell surface properties were measured with the help of the microbial adhesion to solvents (MATS) test and the contact angle measurements (Vatsal et al., 2011). MATS compares the microbial cell affinity to the polar and non-polar solvents (Bellon-Fontaine et al., 1996). The polar solvents can act as electron acceptors or as electron donor. For this, both solvents must possess similar components of the van der Waals surface tension. In our study, the electron donor character (EDC) was measured after determining the cell adhesion to chloroform, which is an electron acceptor solvent, and hexadecane, which is a non-polar solvent; while, the electron acceptor character (EAC) represents the cell adhesion to diethyl ether, which is a good electron donor solvent, and hexane, i.e., non-polar solvent (Bellon-Fontaine et al., 1996; Vatsal et al., 2011). Due to similar van der Waals properties displayed by these solvent pairs, the difference between the values obtained with chloroform and hexadecane and the values measured using diethyl ether and decane indicated the electron donor–electron acceptor interactions occurring at the yeast cell surface and reveals its hydrophilic and hydrophobic properties. The solvent affinity (i.e., percent adherence to every solvent), could be estimated as the proportion of cells which were suspended in these solvents in comparison to the initial cell concentration, with the help of the following equation:

(2) $$Affinity\;or\;adherence(\%)=100\times \left(\frac{1-A}{A_{0}}\right)$$

where, A_0_ and A refer to the A_570_ values of the yeast cell suspension prior and after mixing with the solvent, respectively. The cell surface hydrophobicity (CSH) can be expressed as cells (%) adhered to hexadecane. For eachsolvent, affinity measurements were carried out in triplicates. All solvents were of AR grade and 99% pure according to the manufacturer.

Determination of water contact angle values was carried out with the sessile drop technique with the help of the DigiDrop (GBX, Surface Science technology, France). For this experiment, the cells grown for 72 h on 0.5% (v/v, 47.6 mM) BrB and glucose were mounted on the slides and the surface adhesion energy was determined with the help of the Young’s equation:

(3) $$S=\;\gamma_{1}(\cos\;\theta -1)$$

where, *S* is the surface energy, γ_l_ the surface tension of the liquid and 𝜃 is the contact angle (de Gennes, 1985).

Furthermore, the CFS obtained after growing the cells on BrB (0.5%, v/v; 47.6 mM) and glucose (0.5%, w/v) for 72 h was used for determining the surface tension and the emulsification activity by the method described earlier (Vatsal et al., 2011). The absorbance was measured at 570 nm of the aqueous phase and the absorbance value of 1.0 was defined as 1 U of emulsifying activity (EU/mL).

### BrB Degradation by *Yarrowia lipolytica* NCIM 3589

Concentrations of residual BrB were determined quantitatively by capillary gas chromatography (GC). The CFS collected (20 mL) at the end of every time point (0–96 h) was extracted with 2 volumes of diethyl ether, the sample was concentrated and then analyzed by capillary GC. 1 μl sample (split injection ration; 30:1) was injected on the RTX-5 column (30 m × 0.25 mm × 0.25 μm, Restek, United States), on a Shimadzu 2014 GC (Shimadzu, Japan) with a flame ionization detector, with nitrogen as the carrier gas. The initial temperature of 40°C, was held for 5 min, and then increased to 220°C at the rate of 10°C/min and then held further for 1 min. We also plotted calibration graphs for different BrB concentrations and the residual BrB levels ascertained. The retention time for standard BrB compound was 11.16 min, with the peak being well-resolved under the above conditions. The percent BrB degraded was determined as:

(4) $$C_{control}\;-C_{sample}/C_{control}\;\times \;100\%$$

where *C*_control_ is the concentration of BrB in the media flasks without yeast (i.e., abiotic controls), while C_sample_ is the concentration of BrB in the media flasks with the culture.

For BrB, the degradation rate followed the first order growth kinetics and was determined as follows:

(5) $$l_{n}(C)=-kt+l_{n}(C_{0})$$

where, *C* is the final BrB concentration, C_0_ is the initial BrB concentration, T is the time (per day) while the degradation rate constant can be represented by k (Okpokwasili and Nweke, 2005).

### Bromide Analysis and Enzyme Assays

In this study, the CFS obtained after growing the cells on 0.5% BrB (47.6 mM), as stated above, was used for determining the bromide ions and the dehalogenase activity. Liberation of bromide at 460 nm was measured colorimetrically according to the method described by Iwasaki et al. (1952). Dehalogenase activity was determined by the method by Jesenska et al. (2000) with minor changes. The CFS (100 μL) was incubated with the substrate, i.e., BrB (5 mM) in 50 mM Na-acetate buffer (pH 4.5, 1.8 mL) at 30°C for 30 min and terminated by adding 25 μL of 3 M H_2_SO_4_. One unit (U) of dehalogenase activity could be defined as the amount of enzyme that catalyzed the formation of 1 nmole of bromide per min (Vatsal et al., 2015). All experiments were repeated thrice and the values expressed here are the mean of the three experiments.

Furthermore, intracellular catechol 1,2 dioxygenase and the catechol 2,3 dioxygenase activities were measured according to Hupert-Kocurek et al. (2012).

### Identification of Metabolites

For detection of the metabolites formed due to BrB degradation, initially the glucose-grown *Y. lipolytica* cells were harvested after 24 h of growth. Thereafter, the cells were centrifuged to collect the cell pellet (8000 × *g* at 4°C for 10 min). This pellet was then re-inoculated in 100 mL flasks containing 20 mL of MMY, with 0.5% BrB (47.6 mM) as the substrate. Control flasks containing MMY and 0.5% BrB and no cells were also included. These flasks were then incubated for 48 h; the CFS was collected, concentrated with lyophilisation and then subjected to HPLC. The HPLC analysis was carried out on a C18 column, namely, Poroshell 120 EC (50 mm × 4.6 mm × 2.7 μm) connected to 1260-Quat pumps and a1260 VWD (1260 Infinity, Agilent Technologies, Germany) at 254 nm. Compounds and standards were eluted at the flow rate of 0.7 mL/min with a mobile phase of water–acetic acid (99.5:0.5%, v/v) as solvent A and solvent B as solvent A: acetonitrile (20:80%, v/v).

LCMS was carried out on a Thermo Scientific Q Exactive^TM^ quadrupole-Orbitrap mass spectrometer associated with Accela 1250 pump and Accela open AS. The samples were loaded on a Thermo Scientific Hypersil Gold column (50 mm × 2.1 mm) with a particle size of 1.9 mm. MS and MS/MS experiments were carried out in an ESI-negative ion mode with the following method: sheath gas flow rate 45, auxiliary gas flow rate 10, sweep gas flow rate 2, spray voltage (|KV|) 3.60, spray current (μA) 3.70, capillary temperature (°C) 320, s-lens RF level 50, heater temperature (°C) 350. The ESI–MS data was recorded in a full scan mode for the mass range, *m/z* of 66–1000. The samples were diluted 1:20 and 2 μL samples were injected. The solvents used were methanol and water with 0.1% formic acid at the flow rate of 0.3 mL/min.

The presence of phenol was also confirmed by extracting 1 mL of the sample with a mixture of acetic acid and dichloromethane (1:10, v/v) and GCMS analysis done on a Trace-Ultra GC (Thermo Scientific, United States) with a DB-5 column (30 m × 0.25 mm × 0.25 μm) (Agilent Technologies, United States) and a mass detector (ITQ 1100, Thermo Scientific, United States) with helium as the carrier gas. The column temperature at 60–160°C was increased at the rate of 15°C/min to 200°C at the rate of 3°C/min and held for 1 min. The peaks were identified based on both the *m/z* values and comparison of retention times with standards.

For detecting carbon dioxide, cells were inoculated in sealed serum bottles (60 mL) containing 15 mL MMY with BrB and incubated at 30°C for 120 h at 150 rpm. The headspace in the bottles (inoculated and uninoculated control) was checked for CO_2_ using a GC (Chemito 3800, India) equipped with thermal conductivity detector (TCD) and packed Porapak Q column (stainless steel, 80/100 mesh, 6 feet × 1/8 inch, Sigma-Aldrich, United States) with argon as the carrier gas (flow rate of 40 mL/min). The temperature of the column was 40°C, with the injector and detector temperatures at 70 and 100°C, respectively (Bhadbhade et al., 2002). As a reference standard, carbon dioxide from the gas cylinder containing 80:20 hydrogen:carbon dioxide (mixed gas cylinder, BOC, India) was used.

### Labeled Water Experiments

For confirming that the phenol (Metabolite 1) formed was as a result of the hydrolytic dehalogenation reaction, experiments using labeled water (H_2_O^18^) were carried out. For this, resting cells as well as CFS (100 μL) from above as enzyme source were incubated with BrB (5 mM) in 100 mM Na-acetate buffer (pH 4.5, 1 ml) and an equal volume of labeled water (H_2_O^18^) at 30°C for 2 h. The samples were then centrifuged and supernatant subjected to UPLC–MS or extracted and subjected to GCMS as mentioned above.

LCMS was carried out on a Waters XEVO^TM^ TQD model (Waters, United States) associated with the quadrupole analysers (MS2). The standard and samples were diluted with methanol (1:1) and 10 μL injected on a C18 column of dimensions 100 mm × 2.1 mm × 1.7 μm (Acquity, Waters, United States). Compounds and standards were eluted at the flow rate of 0.7 mL/min, where the mobile phase was water–acetic acid (99.5:0.5%, v/v) as solvent A and solvent B was solvent A: acetonitrile (20:80%, v/v) as mentioned above. MS/MS experiments were carried out in an ESI-positive ion mode using the cone voltage 5 V and collision energy of 3 V. ESI–MS data were recorded in full scan mode. For GCMS, analysis was carried out on a GC 7890 with a HP5 column (30 m × 0.25 mm × 0.25 μm, Agilent Technologies, United States) and MS 7000 (Agilent technologies, United States) as mass detector. All other conditions were similar to those mentioned above.

### Statistical Data Analysis

All the experiments have been carried out in three separate sets where every set has been replicated thrice. The data is expressed as average ± SEM (standard error of mean). A statistical analysis technique of ANOVA (analysis of variance) was also used along with the *F*-test with the help of Mathstat. All values for *p* that were ≤ 0.05 were considered to be significant.

## Results

### Growth on Brominated Aromatic Compounds

To study the degradation and growth on brominated aromatics, two marine strains of *Y. lipolytica* NCIM 3589 and 3590 were selected. *Y. lipolytica* 3589 is a tropical marine strain isolated from the oil polluted regions off Mumbai high with an optimal growth temperature of 30°C while *Y. lipolytica* 3590 (NCYC 789) is a psychotropic marine isolate obtained from Scottish seawaters and has an optimal growth temperature of 20°C (Zinjarde and Pant, 2002). As seen from **Table 1**, *Y. lipolytica* 3589 could grow to differing degrees on MMY containing BrB, 1,2 and 1,3-dibromobenzene and 1,3,5-tribromobenzene with maximal growth on BrB (1.47 g/L). Growth was low on1,2-dibromobenzene, 1,3-dibromobenzeneand 1,3,5-tribromobenzene at 0.1 g/L in 72 h. No growth was seen on 1,4-dibromobenzene and epibromohydrin. *Y. lipolytica* 3590 exhibited a low growth on BrB (0.1 g/L) but failed to grow on any of the other di- or tri-bromobenzenes in 72 h. Thus, it is to be noted that an increase in a number of bromide groups, as well as their position on the ring molecule, affected growth of the yeast. Based on the above studies, *Y. lipolytica* 3589 was selected for further studies. Both these strains have been maintained in the laboratory for over 2 years.

**Table 1:** Cell growth on two strains of *Yarrowia lipolytica* when grown on different bromoaromatics.

Compound^a^	Cell dry mass (g/L)	
	*Yarrowia lipolytica* 3589	*Yarrowia lipolytica* 3590
Epibromohydrin	ND^b^	ND^b^
Bromobenzene	1.47	0.1
1,2-Dibromobenzene	0.1	ND^b^
1,3-Dibromobenzene	0.1	ND^b^
1,4-Dibromobenzene	ND^b^	ND^b^
1,3,5-Tribromobenzene	0.1	ND^b^

*^a^0.5% compound was added to MMY and increase in cell mass for both the strains of *Y. lipolytica* was detected after growth for 72 h.*

*^b^Not detected.*

#### Growth Kinetics on Bromobenzene

**FIGURE 1:**
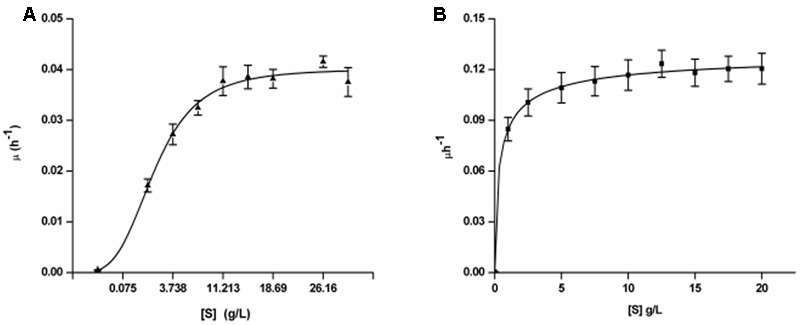
Growth kinetics of *Yarrowia lipolytica* NCIM 3589 on varying initial concentrations of **(A)** BrB; **(B)** Glucose.

Cell dry weights were determined at specific time intervals for various initial BrB concentrations (0–2%, v/v; 0–190 mM). However, very less cell growth was observed for BrB concentrations < 0.1% (v/v, i.e., 9.5 mM) and the cells could grow only at higher BrB concentrations (**Figure 1**). While BrB was not homogeneously dispersed in the medium, it nevertheless supported growth. Hence, a flask was sacrificed at each time point as mentioned in methods to monitor growth.

The specific growth rate (μ) in the exponential phase for every initial substrate concentration (S) was determined with the help of the cell dry weight data and Monod kinetic parameters were calculated by the non-linear regression analyses.

(6) $$\mu =\frac{\mu_{\max}S}{K_{\text{s}}+S}$$

where, μ represents specific growth rate (per h), μ_max_ is maximal specific growth rate (per h), *K_s_* is the BrB concentration at 0.5 μ_max_ (g/L) and S is the initial BrB concentration (g/L).

The specific growth rates (μ) of the exponential phase for every initial BrB concentration (S) was determined and the kinetic parameters were measured using the non-linear regression analyses with the help of the Monod kinetics (Eq. 6). The fit for the growth rate as the function of the initial BrB concentration is shown in **Figure 1** while the kinetic parameters and the *R*^2^ values obtained after carrying out the fits for μ_max_ and *K_s_* is given in **Table 2**. *Y. lipolytica* cells were seen to grow exponentially on various BrB concentrations (0.1–2.0%, v/v; 9.5–190 mM) resulting in the formation of biomass.

**Table 2:** Growth kinetic parameters^a^ for *Yarrowia lipolytica* NCIM 3589 on BrB.

Compound	*R*^2^	μ_max_ (h^-1^)	*K_s_* (g/L)	μ_max_/*K_s_*
Bromobenzene	0.992	0.04	3.26	0.012
Glucose	0.998	0.136	0.426	0.319

*^a^Growth kinetic parameters were estimated after considering the growth of the *Y. lipolytica* cells on BrB and glucose for concentrations ranging between 0.1 and 2% (w/v) using the Monod model.*

When grown on the bromoaromatic compound, it was noted that growth of the yeast was poor at concentrations below 0.1% (v/v, 9.5 mM), while good growth could be seen at higher BrB concentrations (**Figure 1A**). An increase in biomass (as determined by estimating the cell biomass) was seen when the culture was grown on BrB between 1.5 and 29.9 g/L (0.1–2%, v/v). **Table 2** describes the fits for the growth rate as a function of initial BrB concentration and had an *R*^2^ value of 0.992. This high *R*^2^ value suggested that the model fitted well with the experimental data, hence μ_max_ and *K_s_* were determined from the fits. Using the Monod growth kinetics (Eq. 6), the yeast showed a maximal growth rate (μ_max_) of 0.04/h on BrB as compared to 0.136/h on MMY containing glucose. The concentration at which half maximal growth (*K_s_*) was obtained was 3.26 g/L while a specific affinity ratio (μ_max_/*K_s_*) of 0.012 was seen. No cell growth could be seen in the flasks having only MMY media with no BrB or in the flasks with MMY with any yeast cells. Thus, it could be concluded that the yeast was able to grow on BrB as the major carbon source.

On glucose as the major carbon and energy source, the yeast was able to utilize even 0.1% of the glucose and show reasonable growth on low concentrations of the substrate with *R*^2^ value as 0.998 (**Figure 1B**). The cell biomass increased when the glucose concentration increased till concentrations of 5 g/L after which saturation of cell biomass was observed. No growth was observed in medium lacking glucose as the substrate. Using the Monod Kinetic model, the yeast displayed a maximal growth rate of 0.136/h and the concentration where the half maximal cell growth (*K_s_*) was obtained was 0.426 g/L whereas the yeast also showed a specific affinity ratio (μ_max_/*K_s_*) of 0.319 (**Table 2**). Thus, it could be concluded that, though the yeast could utilize glucose very well, *Y. lipolytica* was also able to grow on BrB as the major carbon source.

#### Light Microscopy and Cell Surface Property Studies

**FIGURE 2:**
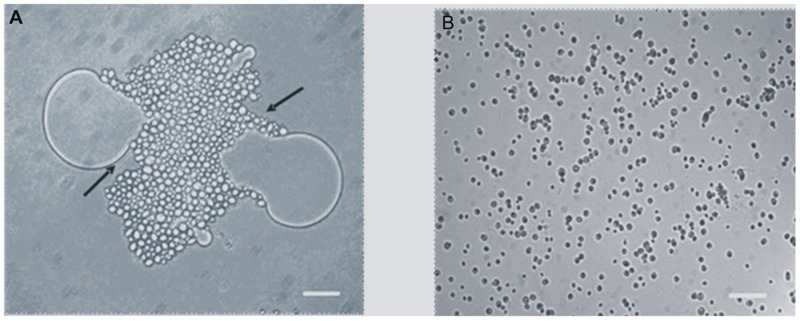
Adhesion of *Y. lipolytica* NCIM 3589 to 0.5% (v/v) of **(A)** Bromobenzene, **(B)** Glucose. Bar represents 20 μm. Arrow indicates the adhesion of the cells to the compound.

When the *Y. lipolytica* cells were grown on 0.5% BrB (47.6 mM), it was seen that the yeast cells adhered to the BrB droplets in the media within 3–4 h, and completely covered the droplets gradually (**Figure 2A**). The glucose grown cells were found to be free and no clumping could be seen (**Figure 2B**).

A significant alteration in the cell sizes in the cells grown on BrB in comparison to those grown on glucose (**Table 3**). The cell size, as determined by the increase in cell diameter, was determined out by taking an average of the cell diameters of more than 25 cells. The mean diameter for the glucose-grown yeast cells was smaller, i.e., 2.28 μm while the diameter of the BrB-grown cells was seen to increase (3.63 μm). A *p*-value of < 0.01 was also noted with the help of the ANOVA test and was considered to be significant. The calculated cell surface area showed a 2.54-fold increase in the cells grown on BrB (41.38 μm^2^) as compared to glucose (16.32 μm^2^).

**Table 3:** Cell surface properties of *Yarrowia lipolytica* NCIM 3589 when grown on bromobenzene and glucose^a^.

Property	Bromobenzene	Glucose
Average cell diameter (μm)	3.63	2.28
Cell surface area (μm)^2^	41.38	16.32
ESV (μm/s)	0.79	0.31
Electron donor capacity (%)	9.97	3.7
Electron acceptor capacity (%)	0.65	-2.29
Cell surface hydrophobicity (%)	89	87.8
Surface tension (mN/m)	48.06	49
Emulsification activity [EU/mL]	0.37	0.33
Water contact angle (𝜃)	51.5	49.1
Surface energy [mN/m^2^]	-27.5	-25.42

*^a^Cells were grown on 0.5% (v/v; 47.6 mM) BrB and glucose for 72 h and further processed as stated in Section “Materials and Methods.” ESV, estimated sedimentation velocity. Electron donor capacity and Electron acceptor velocity were estimated by the MATS test as described in Section “Materials and Methods.” Cell surface hydrophobicity was estimated by MATH test.*

Calculation of Stokes law for determining the estimated sedimentation velocity (ESV) of the yeast cells using the mean cell diameter (**Table 3**). The BrB-grown cells had an ESV of 0.79 μm/s, as compared to cells grown on glucose with an ESV of 0.31 μm/s.

To check whether formation of an extracellular emulsifier or alteration of yeast cell surface properties were responsible for BrB utilization, these parameters were determined and results presented in **Table 3**. In the present study, it can be seen that no change occurred in the surface tension values and even the emulsification activities were similar for the CFS of the cells grown on glucose (0.5%, w/v) and BrB (0.5%, v/v; 47.6 mM).

The Lifshitz-van der Waals electron donor and the electron acceptor characteristics were determined using MATS analysis (Bellon-Fontaine et al., 1996). A majority of the microbial cell surfaces show acid–base interactions, based on their EDC (van Oss, 1993). Strong electron-donating (basic) strains are seen to significantly adhere to chloroform (acidic) as compared to hexadecane, because of their attractive acid–base interactions. Meanwhile, they are also less likely to adhere to ethyl acetate (basic) as compared to decane, because of the repulsive acid–base interactions (Bellon-Fontaine et al., 1996). In our study, the effect of BrB on CSH and acid–base interactions of *Y. lipolytica* 3589 was studied with the help of the MATS test and the water contact angle measurements (**Table 3**). The MATS test compared the relative affinity of the yeast cells grown on glucose and BrB for the monopolar solvent (acidic or basic) and the a polar solvent and all the data has been shown in **Table 3**. Regardless of the medium used, the affinity of the yeast was seen to be always higher on chloroform (i.e., electron acceptor solvent) as compared to hexadecane (i.e., non-polar solvent). The different affinities between these solvents were due to Lewis acid–base interactions (i.e., electron donor–electron acceptor interactions arising from the EDC of the cell surface). The EDC values for BrB (9.97%) were much higher than those on glucose (3.37%) suggestion an increase in the electron donating capacity for the yeast surface when grown in BrB. Likewise, the affinity was similar for diethyl ether (an electron-donating solvent) and decane, with EAC values for both BrB and glucose being low indicating that the electron-accepting nature of the yeast surface is not significant.

The CSH describes the cell potential to attach to hydrophobic and less soluble substrates. The CSH as determined by MATS test was found to be 89 and 87.8% respectively for BrB and glucose suggesting the cell surface to be inherently hydrophobic. The water contact angle measurements are seen to be a better estimate of the CSH (van der Mei et al., 1998). The water contact angle for BrB-grown cells was seen to be 51.5° while that of glucose-grown cells was 49.1°. Since these values were ∼50°, it implies that the yeast cell surface is hydrophobic (**Table 3**). The calculated surface adhesion energy was <0, i.e., -27.5 mN (m^2^)^-1^, which indicated that the yeast cells had a lower affinity for water; which confirmed the general hydrophobic characteristic of the cell surface. To conclude, the presence of BrB in the growth medium did not impart a higher hydrophobicity to the yeast cell surface (as it was inherently hydrophobic) but rather influenced its Lewis acid–base properties.

### Growth and Degradation of BrB by *Y. lipolytica*

To ascertain the probable route in the utilization of the BrB, studies with *Y. lipolytica* were performed where the cells were grown on 0.5% (v/v, 47.6 mM) of BrB. The increase in cell mass resulted in the decrease of the BrB concentration with a simultaneous formation of biomass and release of inorganic bromide ions.

**FIGURE 3:**
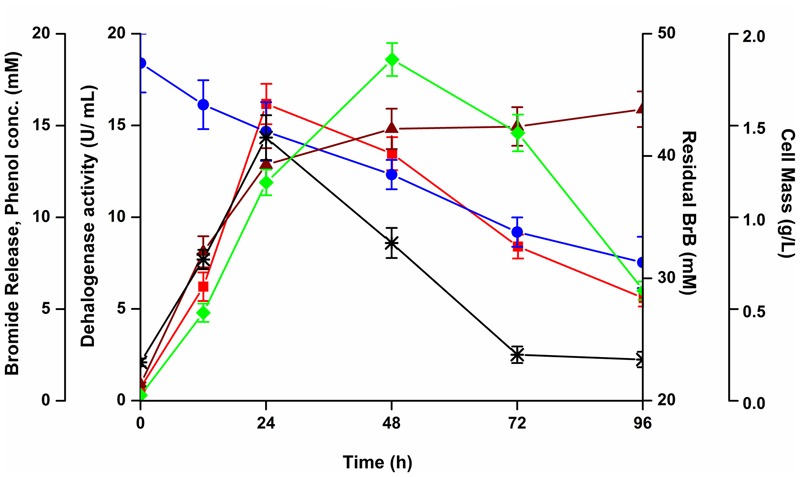
Effect of bromobenzene on the growth, bromide release, degradation and dehalogenase activity of *Y. lipolytica.* The yeast was grown on 0.5% (v/v; 47.6 mM) of bromobenzene in sealed flasks and growth monitored by measuring cell mass (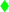), bromide release (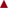), dehalogenase activity (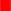), residual bromobenzene (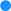), and phenol concentration (^∗^).

The time course of cells grown on 0.5% v/v (7.48 g/L, 47.6 mM) BrB concentration is shown in **Figure 3**. Growth, as seen by an increase in cell dry weight, resulted in the decrease in concentration of BrB and a concomitant release of bromide in the medium. A maximum cell mass of 1.86 g/L was seen within 48 h after which there was a decline in the cell growth. The concentration of bromide released in the medium was determined to be 12.87 mM in 24 h which increased to 15.89 mM in 96 h. The residual BrB concentration was seen to decrease from the initial concentration of 47.6 mM to 31.3 mM at the end of 96 h, i.e., attaining a degradation of 34% with a degradation rate of 0.106 per day. Very less BrB degradation was noted in the abiotic controls lacking yeast cells. The abiotic BrB degradation ranged between 0.3 and 4.1 mM for the time points ranging between 0 and 96 h. The values for the biotic degradation, reported here, refer to the values taken into account after deducting the abiotic degradation for every time point.

A metabolite, which we have identified as phenol (see below) was detected in the media with a maximal concentration of 14.34 mM at 24 h. This metabolite declined thereafter to 2.51 mM in 72 h (**Figure 3**). Thus, the metabolite was being formed as an intermediate during degradation of BrB. The increase in the metabolite concentration in the culture broth coincided with the decrease in BrB concentration, increase in bromide release and higher dehalogenase activity.

### Identification of Metabolites

**FIGURE 4:**
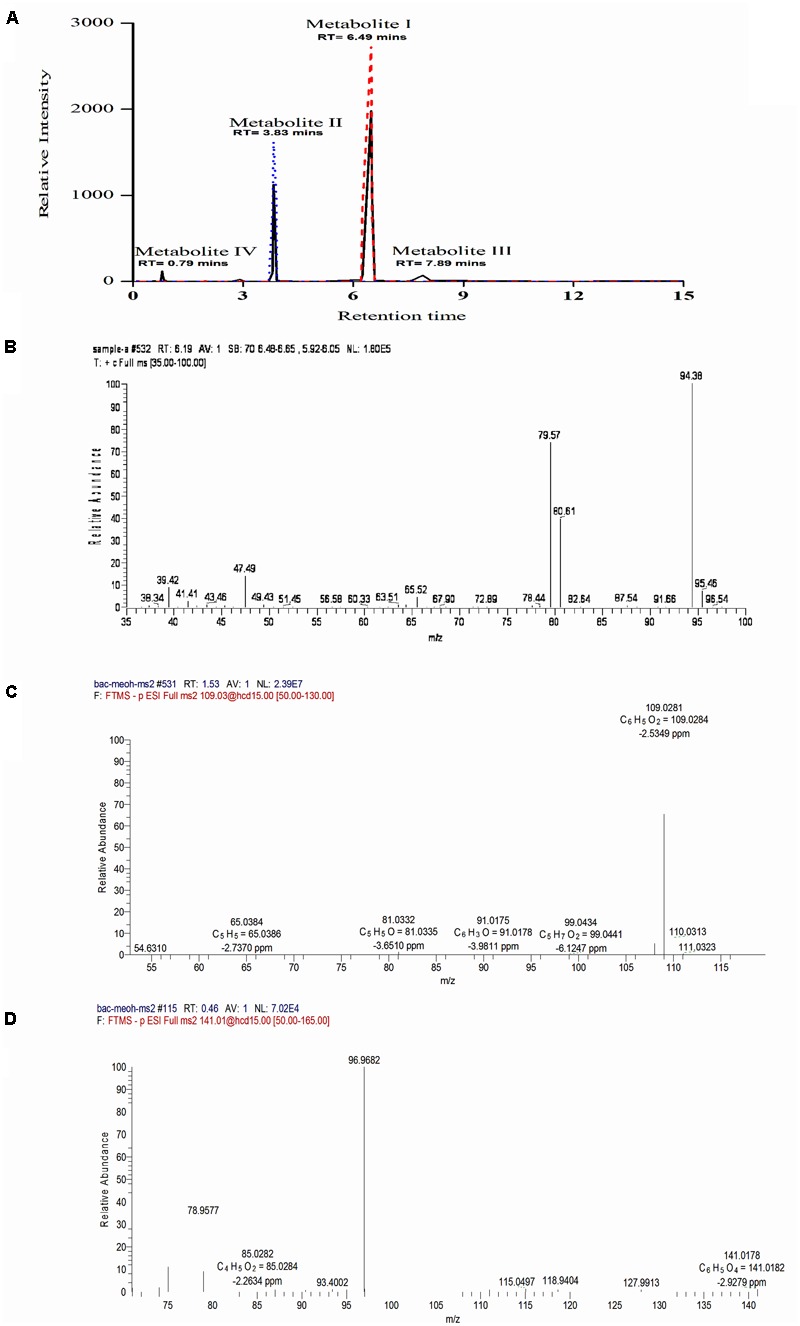
Chromatographic and mass spectrophotometric analysis of the culture supernatant of *Y. lipolytica* NCIM 3589 after growth on bromobenzene. **(A)** HPLC profile of the culture broth extracts (**—**) showing co-elution with the standards, where (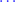) indicates the standard phenol and (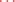) indicates the standard catechol. Metabolite I and II co-eluted with the standard phenol and catechol respectively. **(B)** GCMS spectra for Metabolite I. **(C)** LCMS spectra for Metabolite II. **(D)** LCMS profile for Metabolite IV.

To identify the intermediates or metabolites formed as a consequence of degradation of BrB, studies were carried out under the conditions mentioned in the experimental procedures and samples analyzed by HPLC, LC–MS, and GC–MS. During BrB degradation by the yeast cells, four metabolites (I to IV) appeared in the culture medium in 48 h (**Figure 4A**). Metabolite I, which co-eluted with the authentic phenol standard, was detected with the help of the HPLC (RT = 6.5 min) (**Figure 4A**) and GC (RT = 6.19 min). GC–MS analysis showed that it had a molecular mass of m/z = 94.38 and exhibited fragments at the m/z 94, 80, 79.6, 47 (**Figure 4B**) similar to that of standard phenol. Furthermore, we also identified the Metabolite II as catechol based on the co-chromatography using an authentic catechol standard by HPLC (RT = 3.83 min), and obtained a negative-mode mass spectra of m/z = 109.03 (M^-^H^+^), with fragments at m/z 54.6, 65, 81, 91, 99, 109 (**Figure 4C**). Metabolite III, m/z = 127.1 (M^-^H^+^) could not be identified. Metabolite IV was identified as *cis, cis* muconic acid by mass spectrometry with a m/z = 141.01 (M^-^H^+^) and fragments at m/z 75, 79, 85, 93, 97, 115, 119, 128, 141 (**Figure 4D**).

**FIGURE 5:**
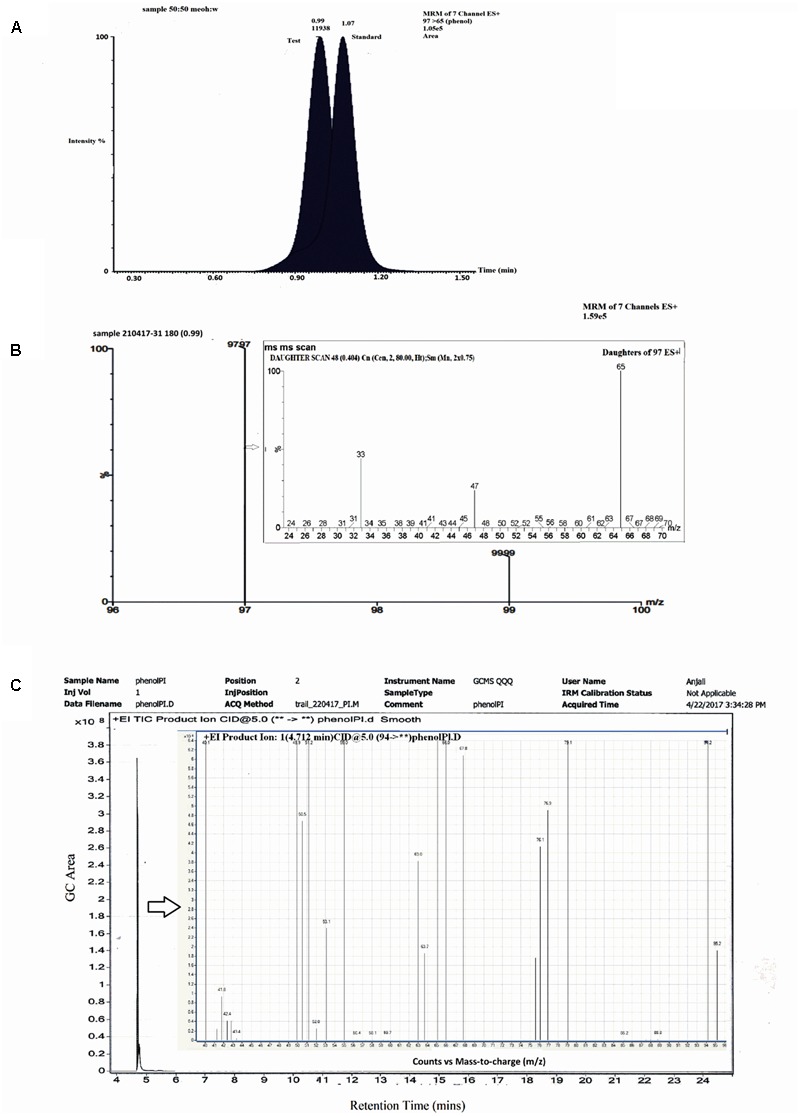
LCMS and GC–MS analysis of labeled water experiments. **(A)** Chromatographic profile of standard phenol and test sample. **(B)** MRM spectra of test sample showing two fragments at m/z 97 and 99. **(B)** Inset: MSMS spectra of test m/z 97. **(C)** GC profile of test sample showing a single peak at Rt 4.712 min. **(C)** Inset: Mass spectra of peak at 4.712 min.

Studies with labeled water (H_2_O^18^) showed the presence of a single peak which eluted at RT = 0.99 min (**Figure 5A**) on LCMS in a positive mode. The standard phenol (m/z = 97) eluted at 1.07 min (**Figure 5A**) with m/z of daughter ions at 65, 47, and 33. The MRM scan of the test sample with labeled water exhibited two m/z values of 97 and 99 with a difference of 2 mass units (**Figure 5B**). MS–MS analysis of the peak with m/z = 97 exhibited daughter ions at m/z = 65, 47, and 33 (**Figure 5B**), similar to that of standard phenol. The peak with m/z = 99 was unable to ionize well under the conditions used and a proper fragmentation profile could not be obtained. The LCMS analysis thereby suggests that incorporation of O^18^ from H_2_O^18^ into phenol is likely. To further confirm the same, analysis was also carried out using GCMS. A single peak eluted at RT = 4.712 min and no other peak could be detected under the experimental conditions used (**Figure 5C**). Ion fragmentation pattern of the peak showed the presence of fragments at m/z = 95.2, 94.2, 79.1, 77, 76.1, 75.3, 67.8, 66, 65, 63.7, 63, 55, 53.1, 52, 51.2, 50.5, 49.9, 41.9, 40.1 (**Figure 5C**, inset). These fragment ion pattern was similar to that of phenol formed from oxygen incorporated from H_2_O while some ion fragments originated from H_2_O^18^, suggesting the incorporation of O^18^ into phenol. Thus, the analysis by both LC–MS and GC–MS suggests that incorporation of O^18^ from the labeled water occurs into phenol by a hydrolytic mechanism.

After analyzing the gas in the headspace of the bottles, we observed that CO_2_ could be detected at the Retention Time (RT) of 2.55 min (GC area = 15265.45) and it was co-eluted with the CO_2_ standard. No significant carbon dioxide was detected in the uninoculated control. The presence of the carbon dioxide in the headspace showed that the BrB could be mineralized by the yeast. Thus, from the above studies, as the initial product formed was via an initial dehalogenation step, the CFS was tested for dehalogenase activity, if any.

### Enzyme Activity in Cell-Free Broth

The release of bromide and presence of phenol as an initial product occurring during cell growth in the above experiments indicates an attack on the ring carbon to which the bromide is attached and suggests that the bromide is being released by a dehalogenation reaction. The CFC collected after the growth of *Y. lipolytica* cells on 0.5% (v/v; 47.6 mM) of BrB was tested for the presence of the extra-cellular debrominating or dehalogenase activity of *Y. lipolytica*.

Maximal extracellular dehalogenase activity of 16.16 U/mL was noted on 0.5% (v/v) of BrB (47.6 mM) after 24 h; which decreased thereafter to 8.4 U/mL at the end of 72 h (**Figure 3**).

No extracellular dehalogenase activity could be detected when glucose was used as the carbon source for growth of the yeast. Similarly, no enzyme activity could be detected in the CFS of cells that were grown on the corresponding products such as phenol or catechol. This suggested that the extracellular dehalogenase enzyme from *Y. lipolytica* was induced in the presence of brominated compounds. A similar observation has been reported when the yeast was grown on bromoalkanes (Vatsal et al., 2015).

The catechol 1,2-dioxygenase and thcatechol 2,3-dioxygenase activities were determined and it was seen that the catechol 1,2-dioxygenase activity was 39.91 nmol/min/mg protein while the catechol 2, 3 dioxygenase activity was much lower at 1.3 nmol/min/mg protein. A higher catechol 1,2 dioxygenase activity provides further evidence that it is a likely route for catechol (Metabolite II) to be degraded via the ortho-cleavage pathway yielding *cis, cis* muconic acid (Metabolite III).

#### Growth of *Y. lipolytica* 3589 on the Products

**FIGURE 6:**
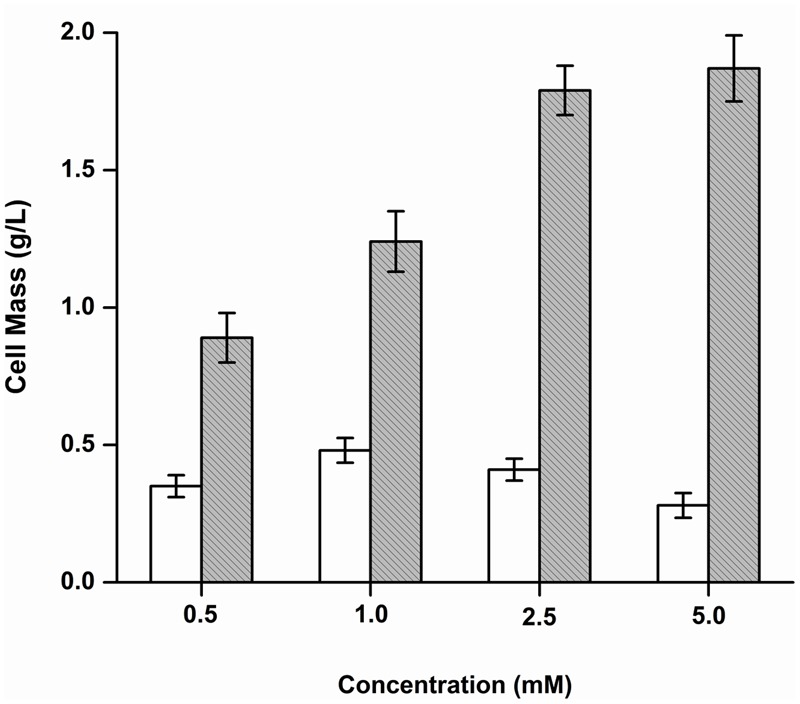
Growth of *Y. lipolytica* on phenol and catechol. Increase in cell mass after growth on phenol (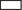) and catechol (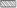) was determined and plotted against different concentrations of the substrates.

The growth of *Y. lipolytica* was also tested in the presence of the various products formed due to the debromination reaction on BrB. It was seen that during growth on the compound, phenol accumulates transiently in the medium attaining a peak concentration of 14.34 mM in 24 h (**Figure 3**). It was also noted that growth and dehalogenation activity decreased after phenol formation. Therefore, it was likely that high concentrations of phenol could be toxic for the yeast. In order to ascertain this, *Y. lipolytica* 3589 was grown on varying concentrations (0.5–5 mM) of phenol and catechol (**Figure 6**). As seen in the figure, a maximal growth of the yeast was obtained on 1 mM phenol which declined at higher concentrations. Also, growth was much lower on phenol as compared to that on catechol. Hence, it is likely that though the yeast is able to tolerate (up to 1 mM) and utilize phenol (as seen by increase in cell mass); a reduction in grow this seen after 48 h (**Figure 3**). Thus, a decrease in cell growth seen after 48 h was most likely due to the presence of high concentrations (>1 mM) of this metabolite which could be toxic to the cells. The yeast is able to tolerate phenol concentrations up to 5 mM as seen in **Figure 5**. In fact, as shown in **Figure 3**, as the phenol concentration reached 14 mM, there was a decrease in dehalogenase activity and a subsequent reduction in cell growth. Thus, the decrease in enzyme activity and cell growth is probably due to the higher concentration of phenol present in the media. On the other hand, good growth on catechol suggests a rapid and effective utilization of the compound by *Y. lipolytica* NCIM 3589.

## Discussion

In this study, we have described the degradation and growth on bromobenzene (BrB), by a tropical marine yeast isolate, *Y. lipolytica* 3589. We have also attempted to identify the metabolites as a result of BrB degradation and proposed a probable pathway for degradation of BrB by *Y. lipolytica*, and this would be of relevance in bioremediation or detoxification of bromoaromatics.

### Growth on the Bromoaromatics and the Mechanism of Adherence

The tropical marine yeast *Y. lipolytica* NCIM 3589 was able to grow well on bromobenzene, an environmentally hazardous compound. Further, it was noted that an increase in a number of the substituent bromide groups, as well as their position on the benzene ring structure, affected the growth of the yeast on other bromoaromatics to differing degrees. The fate and the persistence of several water insoluble aromatic compounds are seen to be dependent on the capability of microbes to use and degrade them. Microbes can either solubilize these hydrophobic compounds using certain surface-active compounds or they could attach to them after altering some of their cell surface properties. Both these phenomena are crucial in understanding the biodegradation and utilization of hydrocarbons by cells (Rosenberg et al., 1980). In the case of *Y. lipolytica*, there has been evidence for both these mechanisms for the hydrophobic compounds (Fickers et al., 2005; Vatsal et al., 2011). To understand the probable mechanism by which *Y. lipolytica* 3589 would be utilizing BrB, both the hypotheses were tested.

When the *Y. lipolytica* cells were grown on 0.5% BrB (47.6 mM), the yeast cells were seen to adhere to the hydrophobic BrB droplet within a short time period (**Figure 2**). We have earlier reported such a phenomenon by the same yeast for *n*-dodecane (Bankar et al., 2009) and bromoalkanes (Vatsal et al., 2011). This fact was significant as it suggested that the bioavailability of the brominated substrate, i.e., BrB would occur due to the attachment of the cells to the hydrophobic droplets. We also observed that there was an increase in the surface area of the cells grown on BrB as compared to those on glucose. Hence, it could be possible that an increased surface area of the cells would lead to a higher attachment of the yeast cells to the hydrophobic BrB droplets; thus, improving its utilization and subsequent debromination. The cells grown on BrB had a higher ESV value as compared to cells grown on glucose. In fact, it was seen that an increasing cell size was directly related to the increased adhesion rate in the yeast *Debaryomyces hansenii*, where a larger cell diameter and a large ESV value resulted in a high adherence of the cells to the substrate (Mortensen et al., 2005). Hence, it could be said that such a similar correlation could exist between the cell size, which led to a high surface area, large ESV and a higher adherence of the *Y. lipolytica* 3589 cells to the hydrophobic substrate, i.e., BrB.

Furthermore, we also determined that there was no change in the surface tension and the emulsification activity, when the yeast cells were grown on the substrate. Thus, a decrease in the surface tension or higher emulsification activity due to production of the extracellular emulsifier is not the strategy employed by *Y. lipolytica* NCIM 3589 for utilizing BrB. Also, we studied the effect of the substrate on the CSH of the *Y. lipolytica*3589 cells with the help of the MATS test and the contact angle measurements. The yeast cells also showed a high CSH when grown on BrB and glucose. We have earlier shown that the strain itself possesses adhering capacity to the hydrophobic substrates (Bankar et al., 2009; Vatsal et al., 2011). Our results were similar to those noted for *Y. lipolytica* 180 where the cells that were grown on glucose and crude oil showed a CSH more than 90% (Kim et al., 1999). Also, the MATS analysis carried out on *Y. lipolytica* IMUFRJ 50682 showed a higher cell adhesion to the hydrophobic compounds (Amaral et al., 2006). The yeast cells grown on BrB exhibited an increase in EDC suggesting that the cell surface of the *Y. lipolytica* 3589 cells display an overall electron donating character. Our results based on the interactions between the cells and BrB show that when *Y. lipolytica* cells were grown on BrB there was no further increase in the CSH, as the cells displayed an inherent hydrophobic surface. However, a small increase in its EDC was noted which suggests that adsorption of the yeast cells to the BrB drop is also due to its electron donating capacity apart from the hydrophobic interactions. Similarly, *Y. lipolytica* W29 cell surface was determined by the MATS test to have much less hydrophobic character while possessing a higher EDC Aguedo et al. (2003).

Based on the light microscopy and cell surface properties of *Y. lipolytica*, when grown on BrB, we conclude that the overall increase in cell size, high CSH and EDC character of cell surface led to an increase in adherence to the BrB droplets enabling its degradation and utilization.

#### Proposed Pathway for Bromobenzene Degradation

**FIGURE 7:**
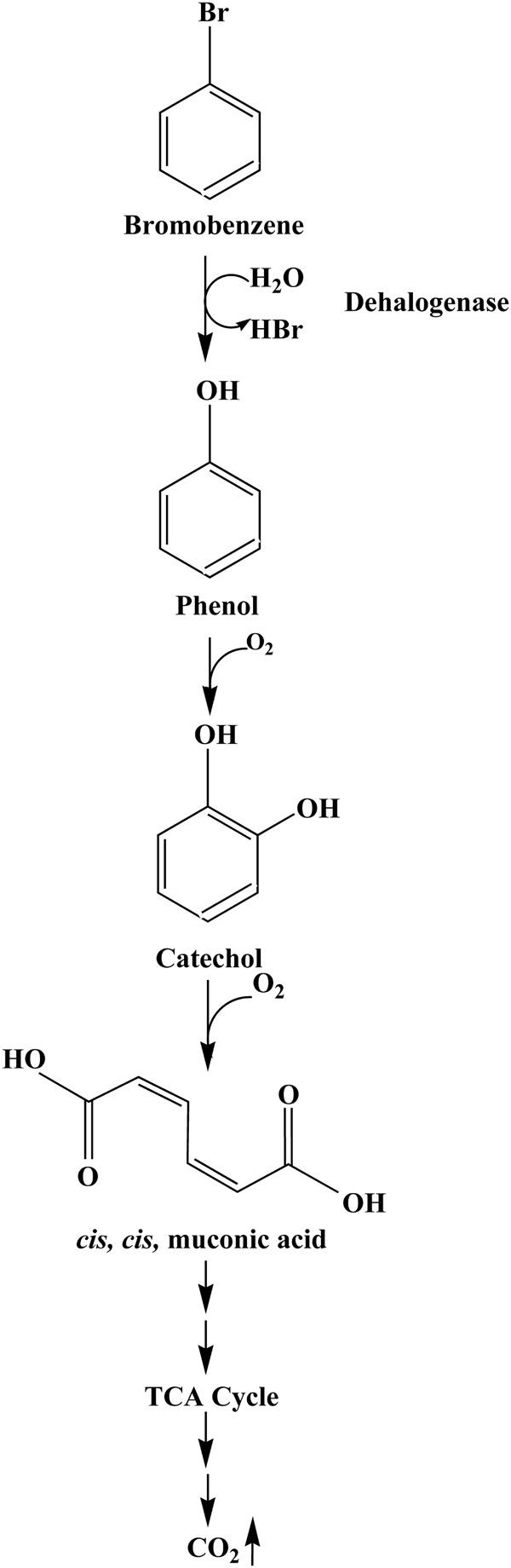
Proposed pathway for BrB degradation by *Y. lipolytica* NCIM 3589.

Based on the metabolites detected, we were able to propose the BrB degradation pathway by *Y. lipolytica* NCIM 3589 in **Figure 7**. The initial step for BrB degradation is a hydrolytic debromination reaction leading to the formation of phenol which is being reported for the first time. In this study, we noted a correlation between cell growth (determined by increasing cell mass), decreasing BrB concentration, release of bromide ions, dehalogenase activity and the phenol appearance, which showed that *Y. lipolytica* NCIM 3589 could degrade BrB, and form phenol as the early intermediate. The mass spectra analysis indicates that phenol is the early intermediate formed during BrB degradation and the further catabolic route is via the ortho cleavage pathway which is generally the pathway followed for phenol degradation by the yeasts. Furthermore, the occurrence of phenol as a metabolite in the early step of the pathway implies that degradation of BrB was initiated by dehalogenation of the carbon atom to which the bromide is attached, i.e., debromination occurs prior to ring cleavage. This was also confirmed by carrying out labeled water experiments, which showed that the resulting phenol was formed as a result of the hydrolytic dehalogenation (**Figure 5**). We were unable to detect any bromocatechol as an early intermediate in this study and the absence of any halocatechol intermediates clearly distinguishes the BrB degradation pathway by *Y. lipolytica* NCIM 3589 from the other chloro- and fluorobenzene catabolic pathways, which proceed via the formation of chloro- and fluorocatechols in *Pseudomonas putida* GJ31 and *Rhizobiales* strain F11 respectively (Mars et al., 1997; Carvalho et al., 2006).

Further metabolism occurs via formation of catechol which is degraded to *cis, cis* muconic acid by the ortho- pathway. It is to be noted that a higher catechol 1,2-dioxygenase activity suggest that this is the likely route for catechol degradation. The appearance of CO_2_ as the final product showed that the organism was able to mineralize the aromatic compound. We were unable to detect any dehalogenase activity when phenol or catechol was used as a sole carbon source for the cell growth experiments. This suggested that the extracellular dehalogenase enzyme from *Y. lipolytica* NCIM 3589 was an inducible enzyme, which could not be detected in the absence of BrB. This suggests an alternative route for degradation of bromoaromatics via an early debromination step, thereby by-passing the formation of bromocatechol whose degradation requires specialized enzymes.

Prior studies on microbial degradation of chloro- and fluorobenzenes have demonstrated that their degradation was initiated by the dioxygenase enzyme and a dihydrodiol dehydrogenase, resulting in the formation of chloro- or fluorocatechols, which were then degraded by the halocatechol pathway resulting in the Krebs cycle intermediates (Pieper et al., 2010). Most strains degrade chlorocatechols via the *ortho*-cleavage pathway, but *meta-*cleavage of 3-chlorocatechol can also occur (Mars et al., 1997; Pieper et al., 2010). Only in some cases such as in *P. putida* strain CLB 250, mutants of *Pseudomonas* strain B13, and *Alcaligenes eutrophus* B9 dehalogenation and dioxygenation occur concomitantly prior to ring cleavage leading to catechol formation (Engesser et al., 1980; Engesser and Schult, 1989) and the involvement of a halobenzoate dehalogenase has been reported for *Aureobacterium* sp. strain RHO25 and *Pseudomonas* sp. CBS3 and also an *Arthrobacter* sp. (Oltmanns et al., 1989). Chloro-, bromo- and 1, 4-dihalogenated benzenes were degraded to their respective halocatechols by the ortho cleavage pathway in bacteria such as *Pseudomonas chlororaphis* RW71, *Pseudomonas* sp. strain JS150 and *P. putida* (Spain and Nishino, 1987; Haigler et al., 1992; Potrawfke et al., 1998). Similarly, fluorobenzene was also degraded by *Rhizobiales* by cleavage of 4-fluorocatechol via the ortho-pathway (Carvalho et al., 2006). To date, few bacteria such as *Rhodococcus* sp. (Zaitsev et al., 1995), *Pseudomonas* sp. (Sperl and Harvey, 1988), *Planococcus* sp. strain ZD22 (Li et al., 2006) and *Burkholderia fungorum* FLU100 (Strunk and Engesser, 2013) have been shown to grow on bromobenzenes, but information on their degradative products is not available. To the best of our knowledge, *Y. lipolytica* NCIM 3589 is the first yeast, which can grow aerobically and degrade BrB with the formation of phenol as the initial degradation product by a dehalogenation step.

## Conclusion

Bromobenzene being hydrophobic and poorly miscible in water has a reduced bioavailability. The strategy used by the yeast cells to utilize the hydrophobic compound would be by cell adherence to these hydrophobic droplets based on its hydrophobic cell surface. We have suggested the probable degradation pathway of BrB by *Y. lipolytica* and changes in cell surface properties, notably in cell size and EDC. To the best of our knowledge, we have reported for the first time the utilization of a bromoaromatic compound by tropical marine yeast. The initial attack on BrB led to a transient accumulation of phenol as the early intermediate, which occurred by the dehalogenation step/reaction. The degradation pathway followed further is through the formation of catechol and further ring fission of catechol occurs through the ortho cleavage pathway via *cis, cis* muconic acid, to Krebs cycle intermediates eventually leading to CO_2_ production. *Y. lipolytica* 3589 was also able to utilize both phenol and catechol as sources of carbon. Since BrB is a compound of considerable environmental interest due to its recalcitrance and toxicity, its degradation by *Y. lipolytica* 3589 presents a novel and promising tool for biotransformation or bioremediation of bromoaromatic pollutants.

## Author Contributions

AV conducted all the laboratory experiments and drafted this manuscript. SZ helped in drafting the manuscript. AR directed the study, coordinated and compiled the data and drafted the manuscript. All the authors have read and approved this final manuscript.

## Conflict of Interest Statement

The authors declare that the research was conducted in the absence of any commercial or financial relationships that could be construed as a potential conflict of interest.
